# Parallel Mapping and Catheter Ablation of Polymorphic Premature Ventricular Contractions: A New Feature for Activation Mapping

**DOI:** 10.1155/2021/9959158

**Published:** 2021-10-08

**Authors:** Moritz Nies, Ruben Schleberger, Leon Dinshaw, Andreas Rillig, Andreas Metzner, Christian Meyer

**Affiliations:** ^1^Department of Cardiology, University Heart and Vascular Center, University Medical Center Hamburg-Eppendorf, Martinistraße 52, 20246 Hamburg, Germany; ^2^DZHK (German Center for Cardiovascular Research), Partner Site Hamburg/Lübeck/Kiel, Berlin, Germany; ^3^Division of Cardiology, EVK Düsseldorf, cNEP, Cardiac Neuro- and Electrophysiology Research Consortium, Kirchfeldstraße 40, 40217 Düsseldorf, Germany; ^4^cNEP, Cardiac Neuro- and Electrophysiology Research Consortium, Institute for Neural and Sensory Physiology, Heinrich Heine University Düsseldorf, Medical Faculty, Düsseldorf, Germany

## Abstract

We report the case of an 80-year-old female presenting with polymorphic premature ventricular contractions, nonischemic cardiomyopathy, and severe, secondary mitral regurgitation. Despite a low intraprocedural PVC burden, activation mapping and successful ablation of different morphologies were achieved using a novel mapping tool, which facilitates simultaneous mapping of different PVC morphologies.

## 1. Introduction

For symptomatic patients with a high burden of polymorphic premature ventricular contractions, catheter ablation is a viable treatment option (1). So far, during activation mapping with a three-dimensional electroanatomical mapping system, only PVCs matching the pattern of the currently active map are registered. Other morphologies have to be addressed sequentially in separate mapping approaches, challenging the effective treatment of polymorphic PVCs with low intraprocedural prevalence. A novel algorithm (CARTO III Software Version 7, Carto Prime, Biosense Webster, Diamond Bar, CA, USA) facilitates simultaneous mapping of different morphologies and offers enhanced mapping opportunities in cases with rare, polymorphic PVCs.

## 2. Case Presentation

### 2.1. History of Presentation

We report the case of an 80-year-old female who presented to our outpatient clinic with recurrence of polymorphic PVCs, resulting in palpitations and dyspnea. Preexisting medical conditions included dilated, nonischemic cardiomyopathy with a reduced left ventricular ejection fraction of 40%. Furthermore, a severe, secondary mitral regurgitation was observed in transthoracic echocardiography. Apart from arterial hypertension and grade I obesity (BMI 31), no cardiovascular risk factors were identified (2).

After unsuccessful treatment with beta-blockers (bisoprolol, nebivolol), two ablation procedures of PVCs had been performed before: ablation in the right ventricular outflow tract and right coronary cusp (2018, initial reduction of PVC-burden from 32% to <1%) and ablation from the left coronary cusp and distal great cardiac vein (2019) had led to suppression of initially predominant morphologies. However, previous low-frequency PVCs with different morphologies had gained in burden. On presentation to our outpatient clinic, Holter-ECG documented a high burden of polymorphic PVCs (52%).

### 2.2. Differential Diagnosis

Aside from idiopathic dilated cardiomyopathy, two reasons were identified as potential causes for the aforementioned left ventricular dilation: first, PVCs in a clinically significant number may cause an arrhythmia-induced cardiomyopathy, mimicking dilated cardiomyopathy (3). Second, severe mitral regurgitation might lead to volume overload and thus to heart failure and dyspnea (4).

### 2.3. Ablation Procedure

Conscious sedation with fentanyl and propofol led to an immediate decrease in the prevalence of PVCs, refractory to provocation with orciprenaline, and after cessation of sedative agents.

**Figure 1:**
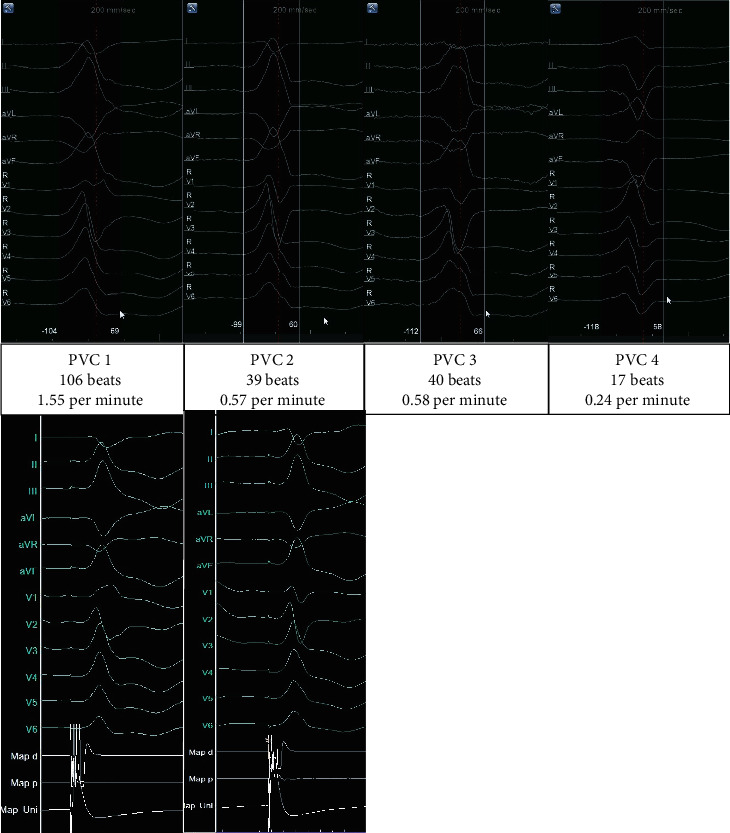
Mapped morphologies. Templates of mapped morphologies of premature ventricular contractions (upper panels), intraprocedural burden, and corresponding pacemaps (lower panels), 200 mm/sec.

With most PVCs showing a morphology indicating a left ventricular origin (Figure 1), a retrograde left ventricular access via the right femoral artery was established. This access proved sufficient for mapping and ablation in this case.

Clinical PVCs remained scarce during the procedure, which is evident in the low number of PVCs (*n* = 202) registered during a total mapping time of 68 : 23 minutes (2.9 PVCs per minute).

Parallel activation mapping of different morphologies was performed using a novel mapping software (CARTO 3, Software Version 7, Biosense Webster, Diamond Bar, CA, USA). Considering their low intraprocedural burden, the different morphologies were mapped simultaneously to record a higher number of PVCs for each respective map.

**Figure 2:**
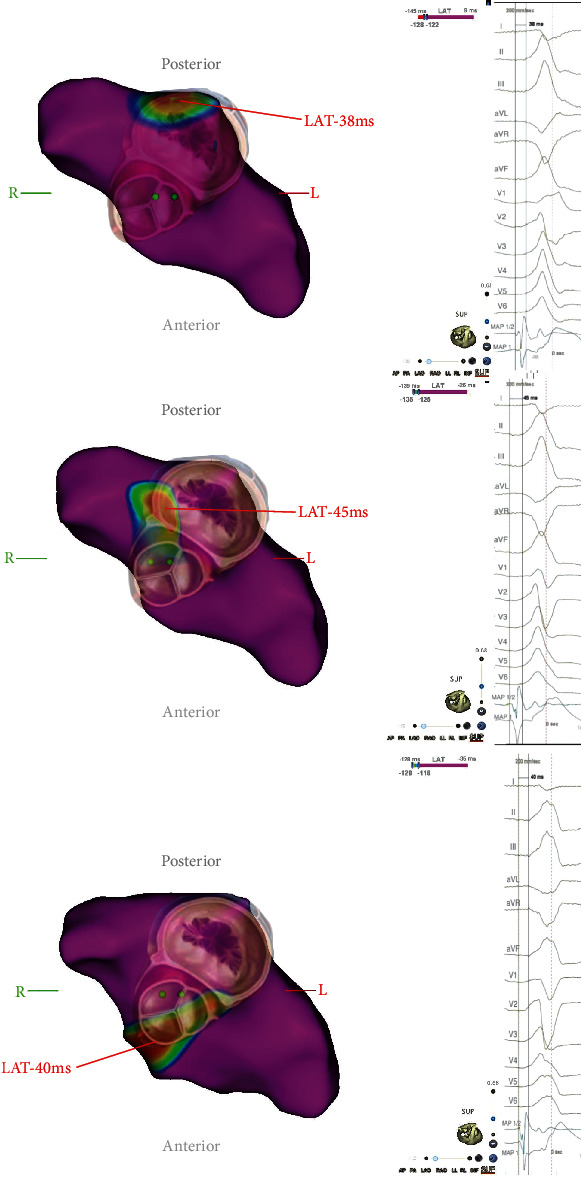
Activation maps. Simultaneously recorded activation maps of polymorphic premature ventricular contractions with corresponding surface ECG and intracardial signals. Partial maps of the left ventricle showing the aortomitral continuity/basal left ventricle, superior view. Color-coded visualization of local activation time (LAT) with the red area indicating the earliest activation. The overlay shows the approximate location of mitral and aortic valve.

Four different morphologies were mapped, three of which were deemed clinically significant. Even though morphologies 2 and 3 (Figure 1) had a very low burden of 0.57 and 0.58 PVCs per minute, the three-dimensional map was sufficient for correct identification of the PVC origin. For PVCs 1-3, the earliest activation was recorded at different locations in close proximity to the aortomitral continuity (AMC, Figure 2). For morphologies 1 and 2, pacemapping was performed and showed a match of 96% (in 12/12 ECG-leads) for both morphologies in their respective area of earliest activation. Regarding morphology 3, several spontaneous PVCs occurred in a short time with an early local activation time prior to ablation. Therefore, the ablation target was viewed as sufficiently localized, and pacemapping was not deemed necessary to confirm its localization while pacemapping can result in slight movements of the catheter itself. Also, to use the PVC disappearance as a success indicator/“procedural” endpoint, any delay to ablation was to be avoided, and no pacemapping was performed.

Consecutive RFC-ablation at the corresponding site of the earliest activation led to acute suppression of all targeted morphologies. To consolidate the ablation lesion for PVC 1, complimentary ablation via the distal great cardiac vein was performed. For mapping and ablation, a 3.5 mm tip catheter was used (Navistar D-Curve, 7 F, Biosense Webster, Diamond Bar, CA, USA). In total, 20 pulses were applied with a cumulative ablation time of 1375 seconds and titration to a maximum energy of 35 Watts for ablation in the left ventricle and 20 Watts for ablation in the distal great cardiac vein. Considering its very low burden (0.24 PVCs per minute) and remote location of origin, PVC was not addressed with catheter ablation in this intervention.

### 2.4. Follow-Up

In a follow-up visit three months after ablation, the patient reported a significant improvement of dyspnea and palpitations. A 24-hour Holter-ECG documented a reduction of the PVC-burden to <1%. The occasional episodes of peripheral edema and intermittent dyspnea were still observed. In light of these residual symptoms, transcatheter mitral valve implantation was performed later on, leading to further improvement of the clinical functional status of the patient. Conventional surgery was not a viable option for this frail patient, and valve calcification had ruled out percutaneous mitral valve repair. During a 9-month follow-up, no clinically significant burden of PVCs was detected. Echocardiography showed a good result after mitral valve replacement and a stable left ventricular ejection fraction of 40% despite treatment of mitral regurgitation.

## 3. Discussion

In patients with polymorphic PVCs and low intraprocedural burden, successful treatment is especially challenging since both of these features are associated with a lower success rate (5, 6). A lack of clinical PVCs during ablation procedure is a well-described phenomenon and can be caused by different factors, such as circadian fluctuations or administration of sedative agents (7, 8).

If activation mapping is impeded by a low intraprocedural burden of PVCs, pacemapping can be a viable alternative, as studies have shown satisfactory results for mapping and ablation relying solely on pacemaps (7). However, activation mapping has a superior spatial resolution compared to pacemapping (9) and is therefore widely accepted as the preferred strategy whenever possible.

With sequential activation mapping, PVCs not matching the singular active pattern are often ignored and lost to mapping, which may further impede the construction of a satisfactory map. In contrast, parallel mapping continuously includes all preregistered PVCs into a corresponding map and thereby simplifies the activation mapping of PVCs with low intraprocedural burden. Furthermore, consecutive activation mapping and ablation of different morphologies can be time-consuming. Parallel mapping might reduce the total procedure and fluoroscopy time as well as the dosage of sedative agents, limiting the risk for associated complications (10).

The feasibility of this approach is supported by the here presented case. However, no final conclusions regarding the usefulness of parallel activation mapping of polymorphic PVCs can be drawn from a single case and further studies are warranted.

The interplay between polymorphic premature ventricular contractions, nonischemic cardiomyopathy, and severe, secondary mitral regurgitation can challenge clinical decision making (11): while polymorphic PVCs are a common finding in patients with congestive heart failure (12) and the burden may increase as a result of a progressing cardiomyopathy (11), they can also be the cause for significant deterioration or development of heart failure (11). This renders the decision difficult which pathology should be treated primarily. Several considerations let to the decision to address the arrhythmia primarily in this case:

Given the high burden of PVC in this case, it was reasonable to suspect that PVCs also affected the evaluation of the functional mitral valve regurgitation, making it appear more severe in this pathologic hemodynamic state. Secondly, the patient's clinical status correlated well with PVC-burden, making this treatment most likely to improve her symptoms. Still, it seemed likely that both pathologies needed to be addressed. For catheter ablation, a transseptal access to the left ventricle might be necessary. Even though small studies have shown the feasibility of this approach after valve intervention (13), this is challenging after mitral valve replacement or percutaneous valve repair. Finally, catheter ablation of PVCs is the less invasive procedure and facilitated more effective cardiac recovery in this case, thereby lowering the risk of the more invasive mitral valve intervention. Considering both options, repeat catheter ablation seemed to be the more reasonable choice for the primary intervention in this case.

## 4. Conclusion

Our case report illustrates that parallel mapping of polymorphic PVCs facilitates simultaneous activation mapping of different morphologies for patients with low intraprocedural PVC-burden, in which the construction of conclusive activation maps might be challenging and time consuming with a sequential mapping approach.

## Data Availability

The clinical and procedural data used to support the findings of this study are available from the corresponding author upon request. The patient provided written consent which is also available upon request.

## Abbreviations

PVC: Premature ventricular contraction
ECG: Electrocardiogram
RFC: Radiofrequency current.

## Additional Points

*Teaching Points*. In conventional, sequential activation mapping of polymorphic PVCs, the construction of the map is limited to one PVC morphology at a time. Other occurring morphologies are lost to the activated map, hampering the localization of ablation targets and prolonging the procedure time. Using a novel mapping software, simultaneous registration of different PVC morphologies is feasible and allows the parallel creation of activation maps of different PVC morphologies. Parallel activation mapping offers the opportunity to successfully localize and ablate the origins of rare polymorphic PVCs for which a sequential mapping approach might not offer sufficient information.
